# Supplementary material for: MetaHiC phage-bacteria infection network reveals active cycling phages of the healthy human gut
Source: eLife. 2021 Feb 26;10:e60608. doi: 10.7554/eLife.60608 (PMC7963479; doi:10.7554/eLife.60608)
Supplement: Transparent reporting form [file elife-60608-transrepform.pdf]

Westbrook Centre
Milton Road
Cambridge CB4 1YG, UK

P 01223 855340
W elifesciences.org
T @elife

eLife's transparent reporting form

We encourage authors to provide detailed information *within their submission* to facilitate the interpretation and replication of experiments. Authors can upload supporting documentation to indicate the use of appropriate reporting guidelines for health-related research (see EQUATOR Network), life science research (see the BioSharing Information Resource), or the ARRIVE guidelines for reporting work involving animal research. Where applicable, authors should refer to any relevant reporting standards documents in this form.

If you have any questions, please consult our Journal Policies and/or contact us: editorial@elifesciences.org.

Sample-size estimation

- You should state whether an appropriate sample size was computed when the study was being designed
- You should state the statistical method of sample size computation and any required assumptions
- If no explicit power analysis was used, you should describe how you decided what sample (replicate) size (number) to use

Please outline where this information can be found within the submission (e.g., sections or figure legends), or explain why this information doesn't apply to your submission:

This work is an exploratory work to test whether meta3C/HiC could be applied to human gut samples or not. We were also wondering what are the results we could obtain regarding interplay between bacteria and phages with this kind of data. Therefore, statistical adaptation of the cohort size does not apply to our study.

Replicates

- You should report how often each experiment was performed
- You should include a definition of biological versus technical replication
- The data obtained should be provided and sufficient information should be provided to indicate the number of independent biological and/or technical replicates
- If you encountered any outliers, you should describe how these were handled
- Criteria for exclusion/inclusion of data should be clearly stated
- High-throughput sequence data should be uploaded before submission, with a private link for reviewers provided (these are available from both GEO and ArrayExpress)

Please outline where this information can be found within the submission (e.g., sections or figure legends), or explain why this information doesn't apply to your submission:

In this study, each of the ten samples was processed through one (7 samples), two (2 samples) or three (1 sample) protocols (meta3C, MetaHiC protocol I or MetaHiC protocol II). For each protocol applied, two independent experiments using a different restriction enzyme were done.
For each protocol and each enzyme, the genomic analysis results were highly concordant (between the different enzymes and between the different protocols). The reads obtained from the different conditions were therefore pooled to increase the resolution of the overall analysis. This is described in the Result section. Given the cost of the experiment, the nature of the protocol, and the depth of the data, no technical replicates were performed.

No data have been removed and all the sample/libraries information could be found in the Fig. 1 and supplementary Files 1 and 2.

All the sequencing data are available one the NCBI database under the following accession: PRJNA627086.

Statistical reporting

- Statistical analysis methods should be described and justified
- Raw data should be presented in figures whenever informative to do so (typically when N per group is less than 10)
- For each experiment, you should identify the statistical tests used, exact values of N, definitions of center, methods of multiple test correction, and dispersion and precision measures (e.g., mean, median, SD, SEM, confidence intervals; and, for the major substantive results, a measure of effect size (e.g., Pearson's r, Cohen's d)
- Report exact p-values wherever possible alongside the summary statistics and 95% confidence intervals. These should be reported for all key questions and not only when the p-value is less than 0.05.

Please outline where this information can be found within the submission (e.g., sections or figure legends), or explain why this information doesn't apply to your submission:

All the statistical methods used in the present studies are reported in the Methods section. P-value are indicated in each corresponding results section.

(For large datasets, or papers with a very large number of statistical tests, you may upload a single table file with tests, Ns, etc., with reference to sections in the manuscript.)

Group allocation

- Indicate how samples were allocated into experimental groups (in the case of clinical studies, please specify allocation to treatment method); if randomization was used, please also state if restricted randomization was applied
- Indicate if masking was used during group allocation, data collection and/or data analysis

eLife Sciences Publications, Ltd is a limited liability non-profit non-stock corporation incorporated in the State of Delaware, USA, with company number 5030732, and is registered in the UK with company number FC030576 and branch number BR015634 at the address Westbrook Centre, Milton Road, Cambridge CB4 1YG.

Please outline where this information can be found within the submission (e.g., sections or figure legends), or explain why this information doesn't apply to your submission:

Our study was performed only using faeces collected from healthy patients and no group has been defined.<br>We received agreement from our Clinical Trial Committee for this study (agreement n°N18 – Ethical statement section).

Additional data files ("source data")

- We encourage you to upload relevant additional data files, such as numerical data that are represented as a graph in a figure, or as a summary table
- Where provided, these should be in the most useful format, and they can be uploaded as "Source data" files linked to a main figure or table
- Include model definition files including the full list of parameters used
- Include code used for data analysis (e.g., R, MatLab)
- Avoid stating that data files are "available upon request"

Please indicate the figures or tables for which source data files have been provided:

We have provided all the data concerning the MAGs obtained in the present study in the supplementary File 4 and 5.<br>The 10 assemblies are also available on the NCBI (PRJNA627086).<br>The code used to generate the data are available on the following github site: https://github.com/mmarbout/HGP-Hi-C

eLife Sciences Publications, Ltd is a limited liability non-profit non-stock corporation incorporated in the State of Delaware, USA, with company number 5030732, and is registered in the UK with company number FC030576 and branch number BR015634 at the address Westbrook Centre, Milton Road, Cambridge CB4 1YG.
